# Supplementary material for: Influence of P3HT:PCBM Ratio on Thermal and Transport Properties of Bulk Heterojunction Solar Cells
Source: Materials (Basel). 2023 Jan 9;16(2):617. doi: 10.3390/ma16020617 (PMC9861154; doi:10.3390/ma16020617)
Supplement: Supplementary file 1 [file materials-16-00617-s001.zip › materials-2084114-supplementary.pdf]

materials

MDPI

Article

Influence of P3HT:PCBM ratio on thermal and transport properties of bulk heterojunction solar cells

Dorota Korte[1,*], E. Pavlica[1], D. Klančar[1], G. Bratina[1], Michal Pawlak[2], EwaGondek[3], Peng Song[4,5], J. Liu[5,6], Beata Derkowska-Zielinska[2]

[1] Laboratory for Environmental and Life Sciences, University of Nova Gorica, Vipavska 13, SI-5000 Nova Gorica, Slovenia

[2] Laboratory for Organic Matter Physics, University of Nova Gorica, Vipavska 13, SI-5000 Nova Gorica, Slovenia

[3] Institute of Physics, Faculty of Physics, Astronomy and Informatics, Nicolaus Copernicus University, 87-100 Torun, Poland

[4] Institute of Physics, Cracow University of Technology, 30-084 Kraków, Poland

[5] State Key Laboratory of Robotics and System, Harbin Institute of Technology, Harbin 150001, China

[6] School of Instrumentation Science and Engineering, Harbin Institute of Technology, Harbin 150001, China

[7] School of Mechatronics Engineering, Harbin Institute of Technology, Harbin 150001, China

* Correspondence: Correspondence: dorota.korte@ung.si

Supplementary Materials

1. Photothermal beam deflection spectrometry – theory

1.1 *Temperature oscillations*

The temperature (ϑ) distribution of i -layer satisfies the Fourier-Kirchhoff equation [58]:

$$\frac{1}{D_i}\frac{\partial \vartheta_i}{\partial t} = \nabla^2 \vartheta_i + \frac{q_i}{k_i}; D_i = k_i/(c_p\rho) \quad \text{(SM.1)}$$

where D_i is thermal diffusivity, k_i, c_p and $\rho$are the material thermal conductivity, heat capacity and density, respectively.q_i is the power density of internal heat sources. These heat sources represent the heat produced by the absorption of the excitation light beam (EB). From here on, it was assumed that EB is only absorbed in the central layer comprising P3HT:PCBM and PEDOT:PSS layers. Other layers exhibit negligible light absorption. Thus, for them the Eq.(SM.1) can be rewritten in a form:

$$\frac{1}{D_i}\frac{\partial \vartheta_i}{\partial t} = \nabla^2 \vartheta_i; \quad \text{(SM.2)}$$

The incident light of EB is modulated with a frequency f in the range between 0 to I_0. As a result, the power density of internal heat sources can be given by [59-64]:

$$q_{si}(z,t) = \frac{\alpha I_0 E_g}{h\upsilon}\left\{\frac{\tau}{2}\left[\nu_{SR} + \frac{1}{\tau}\right] + \left(\frac{h\upsilon}{E_g} - 1\right)[1 + \exp(2i\pi f t)]\right\}\exp(-\alpha z); \quad \text{(SM.3)}$$

where E_g is the energy band gap, τ is the carrier life time, v_{SR} is the surface recombination velocity, α is the effective optical absorption coefficient of the light absorbing layer. Briefly, Eq.(SM.7) accounts for heat generated by (i) intraband thermalization, (ii) bulk and (iii) surface recombination of photogenerated charge carriers in a semiconducting

material [60]. Photogenerated heat is transported to near layers. In fact, the temperature and the heat flux are conserved at the interface between layers, which results in a set of boundary equations:

$$\vartheta_f(z,t)\big|_{z=0} = \vartheta_{s1}(z,t)\big|_{z=0}; \tag{SM.4a}$$

$$\vartheta_{sj-1}(z,t)\big|_{z=l_{sj-1}} = \vartheta_{sj}(z,t)\big|_{z=l_{sj-1}}; \tag{SM.4b}$$

$$\vartheta_{sm}(z,t)\big|_{z\to-\infty} = \vartheta_f(z,t)\big|_{z\to+\infty} = 0; \tag{SM.4c}$$

$$-k_{s1}\frac{\partial\vartheta_{s1}(z,t)}{\partial z}\bigg|_{z=0} = -k_f\frac{\partial\vartheta_f(z,t)}{\partial z}\bigg|_{z=0} \tag{SM.4d}$$

$$-k_{sj-1}\frac{\partial\vartheta_{sj-1}(z,t)}{\partial z}\bigg|_{z=l_{sj-1}} = -k_{sj}\frac{\partial\vartheta_{sj}(z,t)}{\partial z}\bigg|_{z=l_{sj-1}} \tag{SM.4e}$$

A solution of Eqs (SM.1-SM.4) is a set of temperature oscillations in given layers with certain thicknesses $l_{sj}$and thermal properties (D_{sj}, k_{sj}). The temperature oscillation (TOs) in the fluid above the sample is given by:

$$\vartheta_f(z,t) = \theta\exp\left[-\sqrt{\frac{\pi f}{D_f}}(z+z_0)\right]\cos\left[2\pi\pi - \sqrt{\frac{\pi f}{D_f}}(z+z_0)t+\varphi\right]; \tag{SM.5}$$

where θ is the amplitude of TOs at the sample's surface and $\phi$is the phase shift between the phase of the sample surface's temperature change and the phase of the pump beam, which is introduced below. θ and ϕ are functions of thermal (thermal diffusivity and conductivity), optical (energy band gap, absorption coefficient), transport (carrier life time) and structural (thickness) properties of light-absorbing layer and other layers constructing the whole sample (Fig. 1) as described by:

$$\theta = \sqrt{(\mathrm{Re}\theta_m)^2 + (\mathrm{Im}\theta_m)^2}; \quad \varphi = \tan\left(\frac{\mathrm{Im}\theta_m}{\mathrm{Re}\theta_m}\right); \quad \theta_m = U_1\exp\left(l_0\sqrt{i\frac{2\pi\pi}{D_{s1}}}\right) + V_1\exp\left(-l_0\sqrt{i\frac{2\pi\pi}{D_{s1}}}\right); \tag{SM.5a}$$

$$U_1 = \frac{U_2}{\sigma_c}\exp\left(2l_1\sqrt{i\frac{2\pi\pi}{D_{s2}}}\right) + \frac{V_2}{\sigma_c}\exp\left(-2l_1\sqrt{i\frac{2\pi\pi}{D_{s2}}}\right); \; V_1 = U_2\exp\left(2l_1\sqrt{i\frac{2\pi\pi}{D_{s2}}}\right) + V_2\exp\left(-2l_1\sqrt{i\frac{2\pi\pi}{D_{s2}}}\right); \tag{SM.5b}$$

$$U_2 = \frac{1}{\varepsilon_c}\left\{\frac{\eta_{c1}\alpha_{c2}\alpha_{c3}^{-1} - \eta_{c2}}{\eta_{c3} - \alpha_{c1}\alpha_{c3}^{-1}\eta_{c1}}\exp\left[(l_1+2l_2)\sqrt{i\frac{2\pi\pi}{D_{s3}}}\right] + \left(\frac{\varepsilon_{c1}}{\varepsilon_{c3}}\frac{\eta_{c1}\alpha_{c2}\alpha_{c3}^{-1} - \eta_{c2}}{\eta_{c3} - \alpha_{c1}\alpha_{c3}^{-1}\eta_{c1}} + \frac{\varepsilon_{c2}}{\varepsilon_{c3}}\right)\exp\left[-(l_1+2l_2)\sqrt{i\frac{2\pi\pi}{D_{s3}}}\right]\right\} + -\frac{E_3}{\varepsilon_c}\exp[\alpha(l_1+2l_2)]; \tag{SM.5c}$$

$$V_2 = \frac{1}{\varepsilon_c}\frac{\delta_{c1}}{\delta_{c2}}\left\{\frac{\eta_{c1}\alpha_{c2}\alpha_{c3}^{-1} - \eta_{c2}}{\eta_{c3} - \alpha_{c1}\alpha_{c3}^{-1}\eta_{c1}}\exp\left[(l_1+2l_2)\sqrt{i\frac{2\pi\pi}{D_{s3}}}\right] + \left(\frac{\varepsilon_{c1}}{\varepsilon_{c3}}\frac{\eta_{c1}\alpha_{c2}\alpha_{c3}^{-1} - \eta_{c2}}{\eta_{c3} - \alpha_{c1}\alpha_{c3}^{-1}\eta_{c1}} + \frac{\varepsilon_{c2}}{\varepsilon_{c3}}\right)\exp\left[-(l_1+2l_2)\sqrt{i\frac{2\pi\pi}{D_{s3}}}\right] - E_3\exp[\alpha(l_1+2l_2)]\right\}; \tag{SM.5d}$$

$$\sigma_c = \exp\left[\sqrt{i\frac{2\pi\pi}{D_{s1}}}(l_0+l_1)\right]\left\{1+\left(\frac{k_{s1}}{k_f}\sqrt{\frac{D_f}{D_{s1}}}-1\right)\left(\frac{k_{s1}}{k_f}\sqrt{\frac{D_f}{D_{s1}}}+1\right)^{-1}\exp\left[2l_1\sqrt{i\frac{2\pi\pi}{D_{s1}}}\right]\right\}; \; \sigma_{c1} = \sigma_c^{-1}\left(\frac{k_{s1}}{k_f}\sqrt{\frac{D_f}{D_{s1}}}-1\right)\left(\frac{k_{s1}}{k_f}\sqrt{\frac{D_f}{D_{s1}}}+1\right)^{-1}\exp\left[2l_0\sqrt{i\frac{2\pi\pi}{D_{s1}}}\right] \tag{SM.5e}$$

$$\delta_{c1} = \sqrt{i\frac{2\pi\pi}{D_{s1}}}\exp\left[2l_1\sqrt{i\frac{2\pi\pi}{D_{s2}}} - (l_0-l_1)\sqrt{i\frac{2\pi\pi}{D_{s1}}}\right]\sigma_{c1} + \frac{k_{s2}}{k_{s1}}\sqrt{i\frac{2\pi\pi}{D_{s2}}}\exp\left(2l_1\sqrt{i\frac{2\pi\pi}{D_{s2}}}\right) - \frac{1}{\sigma_c}\sqrt{i\frac{2\pi\pi}{D_{s1}}}\exp\left[2l_1\sqrt{i\frac{2\pi\pi}{D_{s2}}} + (l_0-l_1)\sqrt{i\frac{2\pi\pi}{D_{s1}}}\right];$$

$$\delta_{c2} = \frac{1}{\sigma_c}\sqrt{i\frac{2\pi\pi}{D_{s1}}}\exp\left[-2l_1\sqrt{i\frac{2\pi\pi}{D_{s2}}} + (l_0-l_1)\sqrt{i\frac{2\pi\pi}{D_{s1}}}\right] + \frac{k_{s2}}{k_{s1}}\sqrt{i\frac{2\pi\pi}{D_{s2}}}\exp\left(-2l_1\sqrt{i\frac{2\pi\pi}{D_{s2}}}\right) - \sigma_{c1}\sqrt{i\frac{2\pi\pi}{D_{s1}}}\exp\left[-2l_1\sqrt{i\frac{2\pi\pi}{D_{s2}}} + (l_0-l_1)\sqrt{i\frac{2\pi\pi}{D_{s1}}}\right];$$

$$\varepsilon_c = \exp\left[(2l_1+l_2)\sqrt{i\frac{2\pi\pi}{D_{s2}}}\right] + \frac{\delta_{c1}}{\delta_{c2}}\exp\left(-(2l_1+l_2)\sqrt{i\frac{2\pi\pi}{D_{s2}}}\right);$$

$$\varepsilon_{c1}=\frac{\delta_{c1}}{\varepsilon_c\delta_{c2}}\sqrt{i\frac{2\pi\pi}{D_{s2}}}\exp\left[-(2l_1+l_2)\sqrt{i\frac{2\pi\pi}{D_{s2}}}+(l_1+2l_2)\sqrt{i\frac{2\pi\pi}{D_{s3}}}\right]-\frac{1}{\varepsilon_c}\sqrt{i\frac{2\pi\pi}{D_{s2}}}\exp\left[(2l_1+l_2)\sqrt{i\frac{2\pi\pi}{D_{s2}}}+(l_1+2l_2)\sqrt{i\frac{2\pi\pi}{D_{s3}}}\right]+$$
$$\frac{k_{s3}}{k_{s2}}\sqrt{i\frac{2\pi\pi}{D_{s3}}}\exp\left[(l_1+2l_2)\sqrt{i\frac{2\pi\pi}{D_{s3}}}+(l_0-l_1)\sqrt{i\frac{2\pi\pi}{D_{s1}}}\right]$$

$$\varepsilon_{c2}=\frac{E_3}{\varepsilon_c}\sqrt{i\frac{2\pi f}{D_{s2}}}\exp\left[(2l_1+l_2)\sqrt{i\frac{2\pi f}{D_{s2}}}+\alpha(l_1+2l_2)\right]+-\frac{E_3}{\varepsilon_c}\frac{\delta_{c1}}{\delta_{c2}}\sqrt{i\frac{2\pi f}{D_{s2}}}\exp\left[-(2l_1+l_2)\sqrt{i\frac{2\pi f}{D_{s2}}}-(l_1+2l_2)\sqrt{i\frac{2\pi f}{D_{s3}}}\right]+$$
$$\frac{k_{s3}}{k_{s2}}E_3\left[\frac{\tau}{2}\left(\nu_{SR}+\frac{1}{\tau}\right)+\frac{h\upsilon}{E_g}-1\right]\exp[\alpha(l_1+2l_2)]$$

$$\varepsilon_{c3}=-\frac{\delta_{c1}}{\varepsilon_c\delta_{c2}}\sqrt{i\frac{2\pi\pi}{D_{s2}}}\exp\left[(2l_1+l_2)\sqrt{i\frac{2\pi\pi}{D_{s2}}}-(l_1+2l_2)\sqrt{i\frac{2\pi\pi}{D_{s3}}}\right]+\frac{1}{\varepsilon_c}\sqrt{i\frac{2\pi\pi}{D_{s2}}}\exp\left[(2l_1+l_2)\sqrt{i\frac{2\pi\pi}{D_{s2}}}-(l_1+2l_2)\sqrt{i\frac{2\pi\pi}{D_{s3}}}\right]+$$
$$\frac{k_{s3}}{k_{s2}}\sqrt{i\frac{2\pi\pi}{D_{s3}}}\exp\left[-(l_1+2l_2)\sqrt{i\frac{2\pi\pi}{D_{s3}}}\right];$$

$$\eta_{c1}=-\sqrt{i\frac{2\pi\pi}{D_{s4}}}\frac{k_{s4}}{k_{s3}}\exp\left[(l_1+l_2+2l_3)\sqrt{i\frac{2\pi\pi}{D_{s4}}}\right];$$

$$\eta_{c2}=\frac{\varepsilon_{c2}}{\varepsilon_{c3}}\sqrt{i\frac{2\pi f}{D_{s3}}}\exp\left[-(l_1+2l_2+l_3)\sqrt{i\frac{2\pi f}{D_{s3}}}\right]+E_3\left[\frac{\tau}{2}\left(\nu_{SR}+\frac{1}{\tau}\right)+\frac{h\upsilon}{E_g}-1\right]\exp[\alpha(l_1+2l_2+l_3)]$$

$$\eta_{c3}=\frac{\varepsilon_{c1}}{\varepsilon_{c3}}\sqrt{i\frac{2\pi\pi}{D_{s3}}}\exp\left[-(l_1+2l_2+l_3)\sqrt{i\frac{2\pi\pi}{D_{s3}}}\right]-\sqrt{i\frac{2\pi\pi}{D_{s3}}}\exp\left[(l_1+2l_2+l_3)\sqrt{i\frac{2\pi\pi}{D_{s3}}}\right];$$

$$\alpha_{c1}=\exp\left[(l_1+2l_2+l_3)\sqrt{i\frac{2\pi\pi}{D_{s3}}}\right]+\frac{\varepsilon_{c1}}{\varepsilon_{c3}}\exp\left[-(l_1+2l_2+l_3)\sqrt{i\frac{2\pi\pi}{D_{s3}}}\right];\ \alpha_{c2}=\frac{\varepsilon_{c2}}{\varepsilon_{c3}}\exp\left[-(l_1+2l_2+l_3)\sqrt{i\frac{2\pi\pi}{D_{s3}}}\right]-E_3\exp[\alpha(l_1+2l_2+l_3)];$$

$$\alpha_{c3}=\exp\left[(l_1+l_2+2l_3)\sqrt{i\frac{2\pi\pi}{D_{s4}}}\right];\quad E_3=\frac{\alpha I_E E_g}{h\upsilon k_{s3}}\left(\alpha^2-i\frac{2\pi f}{D_{s3}}\right) \tag{SM.5a}$$

where I_E and f are the intensity and modulation frequency of pumb beam, α and E_g are the absorption coefficient and energy band gap of the examined layer, D_f, k_f are the thermal diffusivity and conductivity of air over the whole sample structure, D_{s1}, k_{s1}, l_0 are the thermal diffusivity and conductivity as well as thickness of both glass layers, D_{s2}, k_{s2}, l_1 are the thermal diffusivity and conductivity as well as thickness of nitrogen layer over P3HT:PCBM sample, D_{s3}, k_{s3}, l_2 are the thermal diffusivity and conductivity as well as thickness of P3HT:PCBM material, D_{s4}, k_{s4}, l_3 are the thermal diffusivity and conductivity as well as thickness of ITO layer.

1.2 Photodeflection signal

In BDS technique the sample's surface is periodically heated by a modulated laser beam called excitation beam (EB) [8], what leads to inducing the temperature oscillation (TOs) in the fluid layer adjacent to the sample surface and thus to variation (gradients) in its refraction index, which depends on the optical and thermal properties of the material and fluid above it detected by monitoring the deflection and phase change of a probe beam (PB) propagating above sample close to its surface. One of the main advantages of BDS is the ability to vary the sampling depth by changing the value of modulation frequency f of TOs. This is determined by a quantity called the thermal diffusion length μ_{th}, which defines the distance measured from the surface of the sample into medium that is penetrated by TOs and expressed by:

$$\mu_{th}=(Di/\pi f)^{1/2} \tag{SM.6}$$

where *Di* is the thermal diffusivity of the material, in which TOs propagates (i = s for sample, $i = f$ for fluid over the sample). It turns outthat μ_{th} depends on the material properties. Thus, by changing the TOs penetration depth, it is possible to obtaininformation about the thickness of the material layer at chosen depth determined by μ_{th}.

As a result of the PB interaction with TOs in the fluid above the sample, its trajectory is changed on the refractive index gradients according to the relation [65]:

$$z_1(\xi,\tau) = n_0^2 s_T \int_0^{\tau} (\tau - \tau') \frac{\partial \vartheta_f}{\partial z} d\tau' \ , \qquad \text{(SM.7)}$$

where n_0 is the refracting index of undisturbed fluid, $s_T = (1/n_0)(dn/dT)$ – the temperature coefficient of refractive index (thermal sensitivity), τ is the running complex coordinate along the PB trajectory, ξ is the PB's coordinate in the input plane of the experimental setup ($z = 0$).

The consequence of the change in PB optical path is the change in the PB phase:

$$\Phi_1 = k n_0^2 s_T \int_0^{\tau} \vartheta_f [z(\tau')] d\tau' \qquad \text{(SM.8)}$$

Thus, the PB intensity changes caused by its interaction with TO results in photodeflection (PD) signal that in case of detection by the use of quadrant photodiode (QP) is given by:

$$S_{PD} = 2K_d \left(\int_0^{+\infty} - \int_{-z0}^{0} \right) dz \int_{-\infty}^{+\infty} dy [\mathrm{Re}(a_1) - k\mathrm{Im}(\Phi_1)] I_0 = A_{PD} \cos(\Omega t + \varphi_f + \varphi_{PD}) \qquad \text{(SM.9)}$$

Here K_d is the detector constant, k is the wave number of PB and I_0 is the light intensity of undisturbed PB.
